# Exploring the interplay between mindful eating and self-compassion: insights from three empirical studies and future directions for research

**DOI:** 10.3389/fpsyg.2025.1545056

**Published:** 2025-09-15

**Authors:** Misba Hussain, Rebecca Keyte, Eliza Kalika, Michail Mantzios

**Affiliations:** Department of Psychology, Birmingham City University, Birmingham, United Kingdom

**Keywords:** mindfulness, mindful eating, self-compassion, self-compassion scales, mindful eating scales

## Abstract

**Background:**

Literature on mindful eating explores both mindful eating behaviour and decision-making for mindful eating jointly, which may not necessarily reflect the accurate nature of what mindful eating truly represents. The present research conducted three studies to explore the relationship between BMI, mindful eating behaviour, decision-making for mindful eating, and self-compassion.

**Method and results:**

Using 150 participants, Study 1 examined the correlations between the Mindful Eating Behaviour Scale (MEBS), the Sussex-Oxford Compassion for Self-Scale (SOCS), and their subscales with BMI. Significant positive associations were found between BMI and focused eating, as well as focused eating and hunger and satiety (MEBS subscales) and various facets of self-compassion, but the findings were conflicting, suggested by several measurement limitations. Study 2 aimed to address limitations in the measurement of mindful eating by investigating its association with self-compassion using an alternative scale, the Mindful Eating Behaviour Scale-Trait (MEBS-T) using 152 participants. The findings suggested only BMI was negatively associated with recognising suffering and tolerating uncomfortable feelings (SOCS subscales), but no other significant relationships were found. Study 3 further explored the interplay between self-compassion and mindful eating with 235 participants, utilising the MEBS-T and the original Self-Compassion Scale (SCS), revealing significant positive relationships between sensory attention and non-judgemental awareness (MEBS-T subscales) with common humanity, and mindfulness (alongside non-judgemental awareness and self-kindness), and significant negative relationships between sensory attention and isolation, and non-judgemental awareness and isolation and over-identification.

**Conclusion:**

These findings indicate that associations between mindful eating and self-compassion exist, and specific components of mindful eating, particularly sensory attention and non-judgemental awareness, may play a critical role in fostering a compassionate relationship with oneself—which, for example, in a context of emotional eating and obesity-related stigma propose clear future directions for research and practice but as described in the original Self-Compassion Scale. A critical interpretation of the combined impact and underlying mechanisms in promoting positive eating behaviour change is discussed.

## Introduction

Traditionally, mindful eating is defined as enhancing a healthier relationship with food, whereby individuals eating mindfully are aware of what they are eating moment by moment, overcoming automatic and impulsive reactions around food (e.g., Kristeller, 2015). As such, mindful eating has been used to promote healthier eating behaviours and treat obesity. For example, over the last decade, research has found promoting mindful eating can lead to weight loss, reduced intake of high energy-dense foods, increased diet-related self-efficacy, and decreased maladaptive eating behaviours (e.g., Allirot et al., 2018; Dalen et al., 2010; Hussain et al., 2021a; Hussain et al., 2021b; Timmerman and Brown, 2012). Nonetheless, the definition of mindful eating has been lately subjected to scrutiny, with Mantzios (2021) suggesting that defining mindful eating as a subjective process does not always reflect what mindful eating truly embodies when considered as an all-inclusive mindfulness practice (see Mantzios, 2021 for a review).

Sensory experiences and non-judgement are two components that appear to be central features of mindful eating when seen as behaviour (see Mantzios, 2021 for a summary of features derived from theories and practices). Sensory experiences involve the smell, taste and texture of foods. By focusing on one or more sensory experiences during eating, individuals become fully immersed in the process and more aware of any thoughts and feelings that arise, even if they are unrelated to the sensory experience. They can then respond non-judgementally by redirecting their attention to the sensory aspects of eating, thereby creating a cycle of sustained attention. (Hussein et al., 2017; Kristeller and Epel, 2014; Mantzios, 2023a). Non-judgement is also effective in addressing emotionally related eating behaviours, where emotional regulation is propagated each time attention is redirected to sensory aspects of the eating experience (Barbosa et al., 2020; Dutt et al., 2019; Hussain et al., 2021b). Therefore, the non-judgemental process is what allows one to reiterate the attentional focus to the experience of eating (Mantzios, 2023a).

Hunger and satiety, however, cannot be observed in a similar non-judgemental manner (Mantzios, 2021, 2023a). For example, when one is evaluating or assessing their hunger and satiety, they respond by deciding when to stop food intake, and this can create a conflicting feedback loop in the capability of maintaining a non-judgemental stance (Mantzios, 2023a). Mantzios (2023a) argued that mindful eating behaviour itself defines the true nature of eating behaviour. In contrast, decision-making for mindful eating governs the decisions related to eating, such as evaluating hunger and satiety (or emotional hunger), managing external factors like eating without distractions, and considering serving sizes (Chandon and Wansink, 2011; da Mata Gonçalves et al., 2019). However, these decision-making processes may not accurately reflect the essence of mindful eating, as in mindfulness practices the decision of where and why to meditate does not reflect the nature of the practice. Therefore, distinguishing between mindful eating behaviour and the decision-making process for mindful eating could simplify the field and enhance applied research (Mantzios, 2023b). The mindful eating behaviour scale (Winkens et al., 2018) is a tool that appears to measure both mindful eating behaviour (e.g., focused eating and attentiveness) and decision-making (e.g., hunger and satiety and distraction), which empirically provides an opportunity to explore these separate domains to build on previous theoretical frameworks (Mantzios, 2021, 2023a, 2023b) and interrelations to mindfulness-based constructs, such as self-compassion.

To promote healthier eating behaviours, previous research has focused on the relationship between mindful eating and self-compassion, with self-compassion being conceptualised as a cognitive, affective and behavioural process consisting of five key features: recognising suffering, understanding the universality of suffering in human experience, feeling for the person suffering, tolerating uncomfortable feelings, and acting or being motivated to act to alleviate suffering (Gu et al., 2020; Strauss et al., 2016; see also Neff, 2003). Self-compassion is suggested to support individuals in engaging in healthier eating behaviours as a way of caring for their bodies, acting as a buffer against psychological risk factors associated with unhealthy eating behaviours (e.g., anxiety and stress), as well as minimising body shame and dissatisfaction (Albertson et al., 2015; Berry et al., 2010; Brenton-Peters et al., 2021; Egan and Mantzios, 2018; Hussain et al., 2022; Kelly and Stephen, 2016; Neff, 2003; Rahimi-Ardabili et al., 2018). Previous research implies a complimentary link between mindful eating and self-compassion in enhancing weight loss and promoting healthier eating behaviours in empirical research (Hussein et al., 2017; Shaw and Cassidy, 2020; Shaw and Cassidy, 2022).

Nonetheless, past literature on mindful eating has often failed to assess both mindful eating behaviour and decision-making for mindful eating together. This omission may lead to an inaccurate representation of what mindful eating truly entails, as noted by some researchers (e.g., Mantzios, 2021), but still offers insight for the development of future interventions and potential associations between mindful eating and self-compassion. The first study aimed to provide a novel exploration into the relationship between mindful eating behaviours (i.e., focused eating and attentiveness) and decision-making for mindful eating (i.e., hunger and satiety and distraction) as separate domains with self-compassion.

## Study 1

### Method

#### Participants

To detect a medium effect size of 0.03, with an alpha of 0.05 and power of 0.95, a minimum of 115 participants were required (this also applies to Study 1 and Study 2). A larger sample size was aimed to account for any participant attrition from failure to complete the study (this also applies to Study 2 and Study 3). One hundred and fifty-nine participants were recruited using opportunity sampling via social media platforms (Facebook and X), and the Research Participation Scheme (RPS) at a West Midlands University. Participants recruited via RPS were awarded two-course credits for their participation, whilst those recruited via social media were not compensated. Due to incomplete data, the final sample consisted of 150 participants, with 131 females and 19 males. Participants reported an average age of 24.55 (*SD =* 8.56), and a mean BMI of 25.73 (*SD* = 5.11).

##### Eligibility

Due to the nature of the study, participants were informed via an information sheet and consent form that they were not eligible to participate if they had been diagnosed with an eating disorder, and were also advised not to participate if they felt uncomfortable around the topic of eating behaviours.

### Materials

#### Demographics

Participants were requested to report their gender and age. To calculate BMI, participants also reported their height and weight; with the following formula being used: weight in kg/height in m^2^.

#### Mindful eating behaviour scale (MEBS)

The MEBS is a self-report scale assessing four subscales: (1) focused hunger, (2) hunger and satiety cues (3) eating with awareness, and (4) eating without distraction (Winkens et al., 2018). The scale consists of 17 items, with example items such as “I notice flavours and textures when I’m eating my food” and “I eat automatically without being aware of what I eat.” Responses range from 1 (never) to 5 (very often), with higher scores suggesting greater levels of mindful eating. The scale has been validated in English and Arabic (Fekih-Romdhane et al., 2023; Mantzios et al., 2022). The present study produced alphas of *α* = 0.88 (focused hunger), *α* = 0.88 (hunger and satiety cues), *α* = 0.91 (eating with awareness), and *α* = 0.75 (eating without distraction).

#### Sussex-Oxford compassion for the self-scale (SOCS)

The SOCS is a self-report scale consisting of five subscales: (1) recognizing suffering, (2) understanding the universality of suffering, (3) feeling for the person suffering, (4) tolerating uncomfortable feelings, and (5) motivation to act/acting to alleviate suffering (Gu et al., 2020). The scale contains 20 items, with responses ranging from 1 (not at all true) to 5 (always true), with higher scores suggesting greater self-compassion. Example items of the scale include “I’m good at recognising when I’m feeling distressed” and “When I’m upset, I do my best to take care of myself.” The scale has been validated in several languages, such as Korean, Swedish and German (Brophy et al., 2024; Kim and Seo, 2021; Sarling et al., 2024). The present study produced alphas of *α* = 0.83 (recognising suffering), *α* = 0.91 (understanding the universality of suffering), *α* = 0.89 (feeling for the person suffering), *α* = 0.84 (tolerating uncomfortable feelings), and *α* = 0.90 (motivation to act/acting to alleviate suffering).

### Procedure

Potential participants responded to online advertisements on Facebook, X and RPS, and were directed via a link to the participant information sheet. Those who wished to participate were then directed to a consent form. Upon providing informed consent, participants were presented with the demographic form and the questionnaires, taking approximately 10 min to complete. Once the study was complete, participants were presented with a debriefing sheet, highlighting the purpose of the study, as well as the researcher’s contact details. Ethical approval was granted by the University’s Research Ethics Committee.

## Results

The assumptions for Pearson correlation coefficient of normality, linearity and homoscedasticity were met. Bivariate inter-correlations between BMI, mindful eating, and self-compassion are presented in Table 1. Findings suggest there is a small significant and positive relationship between focused eating and BMI (*r* = 0.166, *p* = 0.043) and recognising (*r* = 0.260, *p* = 0.001), as well as a moderate significant and positive relationship with understanding (*r* = 0.349, *p* < 0.001). A small significant and positive relationship is displayed between hunger and satiety and recognising (*r* = 0.166, *p* = 0.043), feeling (*r* = 0.234, *p* = 0.004), and tolerating (*r* = 0.227, *p* = 0.005). There is also a small significant and positive relationship between awareness and understanding (*r* = 0.279, *p* < 0.001). Due to the differences in age ranges across the three studies, partial correlations on the above variables were also run to control for age. The results found all associations between variables were similar, except the relationship between focused eating and BMI became non-significant. The partial correlation coefficient table can be found in the supplementary materials.

**Table 1:** Bivariate correlations between BMI, MEBS, and SOCS.

	1	2	3	4	5	6	7	8	9
1. BMI									
2. Focused^1^	0.166^*^								
3. Hunger and Satiety^1^	−0.001	0.154							
4. Awareness^1^	0.080	0.330^**^	0.308^**^						
5. Distraction^1^	−0.023	0.162^*^	0.165^*^	0.331^**^					
6. Recognising^2a^	0.028	0.260^**^	0.166^*^	0.155	0.042				
7. Understanding^2b^	0.085	0.349^**^	0.106	0.279^**^	−0.007	0.444^**^			
8. Feeling^2c^	−0.107	0.062	0.234^**^	0.009	−0.004	0.499^**^	0.197^*^		
9. Tolerating^2d^	−0.115	0.087	0.227^**^	−0.015	0.052	0.485^**^	0.194^*^	0.860^**^	
10. Acting^2e^	−0.108	0.143	0.155	−0.074	−0.057	0.318^**^	0.195^*^	0.434^**^	0.338^**^

^*^Correlation is significant at the 0.05 level, ^**^correlation is significant at the 0.01 level.

1 (Winkens et al., 2018) Subscales of the mindful eating behaviour scale;

2 (Gu et al., 2020) Subscales of the sussex oxford compassion for the Self;

2a Recognizing suffering;

2b Understanding the universality of suffering;

2c Feeling for the person suffering;

2d Tolerating uncomfortable feelings;

2e Motivation to act/acting to alleviate suffering.

## Discussion

These findings contribute partially to our understanding of the complex relationship between BMI, mindful eating, and self-compassion, highlighting the importance of further research to explore these associations in greater depth. Findings indicated that focused eating was positively associated with BMI, as well as with recognizing suffering and understanding the universality of suffering. Additionally, awareness, a component of mindful eating behaviour, showed a positive correlation with understanding the universality of suffering. Decision-making in mindful eating was also explored with self-compassion, revealing that hunger and satiety were positively related to recognizing suffering, empathizing with the suffering of others, and tolerating uncomfortable feelings. The relationships between the four mindful eating subscales and various aspects of the self-compassion scale pose challenges in terms of clarity, coherence, and depth of understanding, making it difficult to articulate these intricacies in a logical manner that paves the way for future research and the implementation of effective practices. As such, future research could consider examining the Mindful Eating Behaviour Scale-Trait (MEBS-T; Mantzios, 2023b) to enhance our understanding of how self-compassion may complement the impact of mindful eating, and vice versa.

### Study 2

Study 2 aimed to address the limitations of the scale developed by Winkens et al. (2018) by investigating its potential association with self-compassion using an alternative scale that measures mindful eating behaviour, and not decision-making for mindful eating. Mantzios (2021) defined Mindful Eating Behaviour (MEB) as “the sustained attention to a sensory element of the eating experience (e.g., the taste) and a non-judgemental (or non-evaluative) awareness of thoughts and feelings that are incongruent to the sensory elements of the present eating experience” (p. 369). This definition provides a more robust and precise foundation for research, rooted in empirical evidence and the principles of secular mindfulness. Mindfulness is commonly understood as an awareness that arises from (a) deliberately focusing on the present moment, with (b) a non-judgemental attitude (Kabat-Zinn, 1990). A non-judgemental attitude facilitates attentional self-regulation in mindfulness practices (Bishop et al., 2004; Shapiro et al., 2006), a principle similarly applicable to mindful eating practices where non-judgemental awareness enables “the self-regulation of sensory attention whilst eating” (Mantzios, 2024, p. 2). Furthermore, Mantzios (2023b) introduced the Mindful Eating Behaviour Scale-Trait (MEBS-T) and associated practices, which are congruent with theories of both mindfulness and mindful eating. The MEBS-T has two subscales assessing sensory attention and non-judgemental awareness, aligning with principles found in both mindfulness and mindful eating literature (Mantzios, 2023b). The aim of Study 2, utilising a scale that was developed to be more aligned to mindfulness practice would shed light into the potential association between the MEBS-T and self-compassion.

## Method

### Participants

Participants were recruited via the Prolific (an online platform for participant recruitment), and were compensated £6.60/ph. The sample included 153 participants; however, one participant was removed as their BMI was under 16.5 kg/m^2^ which is severely underweight (Weir and Jan, 2023) and raises concerns of potential eating disorders. The final sample consisted of 111 females and 41 males, with an average age of 39.69 (*SD =* 13.60), and a mean BMI of 26.36 (*SD* = 5.70). For eligibility, please see Study 1.

### Materials

#### Demographics and Sussex-Oxford compassion for the self-scale (SOCS)

For demographic questions and SOCS, please see Study 1 (Gu et al., 2020). For SOCS, the present study produced alphas of recognizing suffering *α* = 0.81, understanding the universality of suffering *α* = 0.82, feeling for the person suffering *α* = 0.84, tolerating uncomfortable feelings *α* = 0.75, and motivation to act/acting to alleviate suffering *α* = 0.89.

#### Mindful eating behaviour scale-trait (MEBS-T)

The MEBS-T is a self-report scale assessing two subscales: (1) sensory attention and (2) non-judgemental awareness (Mantzios, 2023b). The scale consists of 10 items, with example items such as “I focus on what I am eating” and “When I am eating, I overcome unrelated thoughts and/or feelings by focusing on the food and the sensation of eating.” Responses range from 1 (strongly disagree) to 4 (strongly agree) with higher scores suggesting greater levels of mindful eating. The present study produced alphas of *α* = 0.82 (sensory attention) and *α* = 0.82 (non-judgemental awareness).

### Procedure

Potential participants responded to online advertisements on Prolific, and the following procedure follows Study 1.

## Results

The assumptions for Pearson correlation coefficient of normality, linearity and homoscedasticity were met. Bivariate inter-correlations between BMI, mindful eating behaviour scale-trait, and Sussex Oxford compassion for self-scale are presented in Table 2. Findings suggest there is a small significant and negative relationship between BMI and recognising (*r* = −0.267, *p* < 0.001) and feeling (*r* = −0.190, *p* = 0.019). The remaining relationships were non-significant. Due to the differences in age ranges across the three studies, partial correlations on the above variables were also run to control for age. The results found all associations between variables were similar, except the relationship between BMI and motivation to act or acting to alleviate suffering became significant and negative (*r* = −0.183, *p* = 0.024). The partial correlation coefficient table can be found in the supplementary materials.

**Table 2:** Bivariate correlations between BMI, MEB-T, and SOCS.

	1	2	3	4	5	6	7
1. BMI							
2. SA^1a^	−0.102						
3. NJA^1b^	−0.010	0.372^**^					
4. Recognising^2a^	−0.267^**^	0.155	−0.030				
5. Understanding^2b^	−0.067	0.088	−0.136	0.396^**^			
6. Feeling^2c^	−0.190^*^	0.066	0.142	0.346^**^	0.199^*^		
7. Tolerating^2d^	−0.086	0.028	0.035	0.341^**^	0.164^*^	0.751^**^	
8. Acting^2e^	−0.156	0.037	0.101	0.357^**^	0.175^*^	0.824^**^	0.736^**^

^*^Correlation is significant at the 0.05 level, ^**^correlation is significant at the 0.01 level.

1 (Mantzios, 2023b) Subscales of the mindful eating behaviour scale - trait;

1a Sensory attention;

1b Non-judgmental awareness;

2 (Gu et al., 2020) Subscales of the sussex oxford compassion for the self;

2a Recognizing suffering;

2b Understanding the universality of suffering;

2c Feeling for the person suffering;

2d Tolerating uncomfortable feelings;

2e Motivation to act/acting to alleviate suffering.

## Discussion

This study investigated the connections between BMI, the Mindful Eating Behaviour Scale-Trait (MEBS-T), and the Sussex Oxford Compassion for Self-Scale (SOCS), as shown in Table 2. One notable finding was a small negative relationship between BMI and recognising and feeling which pertains to self-compassion. However, no significant relationships were observed between the MEBS-T, and the SOCS.

Whilst Study 2 aimed to shed light on the relationship between mindful eating and self-compassion following Study 1, it did not significantly contribute to our understanding of how these mindfulness-based constructs are related to each other, and with no indications of compatibility for future research and interventions. Further inquiry is necessary to comprehensively clarify the connection between mindful eating behaviour and self-compassion. Previous research has suggested a positive relationship between mindful eating, as measured by various scales and conceptualizations, and self-compassion. However, it is noteworthy that this relationship has been observed with the original self-compassion scale, and at times, without consistency across data and subscales (e.g., Hussain et al., 2022; Kalika et al., 2023; Keyte et al., 2022; Mantzios et al., 2018; Regan et al., 2023). Further research could consider examining the original self-compassion scale (SCS; Neff, 2003) alongside the MEBS-T (Mantzios, 2023b), to enhance our understanding of how self-compassion and mindful eating may complement each other and impact health outcomes and positive behaviour change.

### Study 3

Exploring the interplay between self-compassion and mindful eating holds promise for enhancing our comprehension of how these constructs intersect and influence health outcomes and eating behaviours. Mantzios and Wilson (2015) have posited that the relationship between self-compassion and mindful eating may yield beneficial effects, particularly regarding emotion regulation within interventions. To deepen this understanding, Study 3 examined the original Self-Compassion Scale (SCS) Neff (2003) alongside the Mindful Eating Behaviour Scale-Trait (MEBS-T; Mantzios, 2023b). This comparative approach has the potential to illuminate the nuanced dynamics between self-compassion and mindful eating, shedding light on their combined impact on individuals’ well-being and dietary practices in future research.

## Method

### Participants

Participants were recruited using opportunity sampling via social media platforms (Facebook and X), the Research Participation Scheme (RPS) at a West Midlands University, and Prolific (compensation £6.60 ph) resulting in a higher sample size. The sample included 238 participants; however, two participants were removed as their BMI was under 16.5 kg/m^2^ which is severely underweight (Weir and Jan, 2023) and raises concerns of potential eating disorders, and another participant was removed due to an inaccurate BMI value. The final sample consisted of121 males, 109 females, and five who did not disclose, with an average age of 29.56 (*SD =* 9.76), and a mean BMI of 25.23 (*SD* = 4.91). For eligibility, see Study 1.

### Materials

#### Demographics and mindful eating behaviour scale-trait (MEBS-T)

For demographic questions and MEBS-T, please see Study 1 and Study 2 (Mantzios, 2023b). For MEBS-T study 3 produced alpha’s of *α* = 0.82 (sensory attention) and *α* = 0.85 (non-judgemental awareness).

#### Self-compassion scale (SCS)

The SCS is a 26 self-report item assessing six subscales: (1) self-kindness vs. (2) self-judgement, (3) common humanity vs. (4) isolation, (5) mindfulness vs. (6) overidentification (Neff, 2003). Sample items include “I try to be loving towards myself when I’m feeling emotional pain” and “When I think about my inadequacies, it tends to make me feel more separate and cut off from the rest of the world.” Scores range from 1 (almost never) to 5 (almost always), with higher scores suggesting higher levels of self-compassion. The scale has been validated in several languages, such as Italian, Japanese, and Portuguese (Arimitsu, 2016; Bento et al., 2016; Petrocchi et al., 2014). The present study produced alphas of *α* = 0.84 (self-kindness), *α* = 0.76 (self-judgement), *α* = 0.73 (common humanity), *α* = 0.77 (isolation), *α* = 0.77 (mindfulness), and *α* = 0.75 (overidentification).

### Procedure

Potential participants responded to online advertisements on Facebook, X, RPS, and Prolific, and the following procedure follows Study 1.

## Results

The assumptions for Pearson correlation coefficient of normality, linearity and homoscedasticity were met. Bivariate inter-correlations between BMI, mindful eating behaviour scale-trait, and self-compassion scale are presented in Table 2. Findings suggest there is a small significant and negative relationship between BMI and overidentification (*r* = 0.137, *p* = 0.036). A small significant and positive relationship between sensory attention and common humanity (*r* = 0.182, *p* = 0.005), and mindfulness (*r* = 0.153, *p* = 0.019), and a small and negative relationship between sensory attention and isolation (*r* = −0.196, *p* = 0.003). There is also a small significant and positive relationship between non-judgemental awareness and self-kindness (*r* = 0.209, *p* = 0.001), common humanity (*r* = 0.155, *p* = 0.017), and mindfulness (*r* = 0.137, *p* = 0.035), as well as a negative relationship between non-judgemental awareness and isolation (*r* = −0.148, *p* = 0.023) and overidentification (*r* = −0.142, *p* = 0.029). Due to the differences in age ranges across the three studies, partial correlations on the above variables were also run to control for age. The results found all associations between variables were similar, except the relationship between sensory attention and self-kindness became positive and significant (*r* = 0.142, *p* = 0.031), whilst the relationship became slightly non-significant between non-judgement awareness and isolation (*r* = −0.128, *p* = 0.053) and overidentification (*r* = −0.125, *p* = 0.057). The partial correlation coefficient table can be found in the supplementary materials.

## Discussion

The findings from Study 3 using BMI, the Mindful Eating Behaviour Scale-Trait (MEBS-T; Mantzios, 2023b) and the Self-Compassion Scale (SCS; Neff, 2003) suggest a negative relationship between BMI and overidentification, with both sensory attention and non-judgemental awareness being positively associated with common humanity and mindfulness, and negatively associated with isolation. Additionally, non-judgemental awareness was also found to be positively related to self-kindness and negatively related to overidentification. The findings from this study align with previous literature on the relationship between mindful eating and self-compassion (Hussain et al., 2022; Kalika et al., 2023; Keyte et al., 2022; Mantzios et al., 2018; Regan et al., 2023), and provide some further clarity on the interrelation between mindful eating and self-compassion (see Table 3).

**Table 3:** Bivariate correlations between BMI, MEB-T, and SCS.

	1	2	3	4	5	6	7	8
1. BMI								
2. SA^1a^	−0.024							
3. NJA^1b^	0.035	0.235^**^						
4. Self-kindness^2^	−0.018	0.127	0.209^**^					
5. Self-judgement^2^	−0.091	−0.108	−0.103	0.493^**^				
6. Common humanity^2^	−0.041	0.182^**^	0.155^*^	0.583^**^	0.298^**^			
7. Isolation^2^	−0.057	−0.196^**^	−0.148^*^	0.347^**^	0.669^**^	0.227^**^		
8. Mindfulness^2^	−0.025	0.153^*^	0.137^*^	0.701^**^	0.326^**^	0.528^**^	0.288^**^	
9. Overidentification^2^	−0.137^*^	−0.118	−0.142^*^	0.348^**^	0.684^**^	0.236^**^	0.729^**^	0.379^**^

^*^Correlation is significant at the 0.05 level, ^**^correlation is significant at the 0.01 level.

1 (Mantzios, 2023b) Subscales of the mindful eating behaviour scale-trait;

1a Sensory attention;

1b Non-judgmental awareness;

2 (Neff, 2003) Subscales of the self-compassion scale.

### General discussion

Across three studies, the relationship between BMI, mindful eating and self-compassion was explored. These studies collectively provide insights into how mindful eating and self-compassion are related to BMI and each other, and will be reviewed next.

In Study 1, the Mindful Eating Behaviour Scale (MEBS; Winkens et al., 2018) was used to explore two distinct domains: (1) mindful eating behaviour (e.g., focused eating and awareness) and (2) decision-making related to eating (e.g., hunger, satiety, and distraction). This was examined in conjunction with the Sussex-Oxford Compassion for the Self Scale (SOCS; Gu et al., 2020) and BMI. The findings suggested that mindful eating behaviour (a) focused eating was positively associated with BMI, recognising suffering, and understanding the universality of suffering, and (b) awareness was also positively associated with understanding the universality of suffering. For decision-making the findings proposed that (a) hunger and satiety was positively associated with recognising suffering, feeling for the person suffering, and tolerating uncomfortable feelings. Findings from Study 1 make interpretations for behavioural implications unclear due to the inconsistency in outcomes between mindful eating, decision-making and self-compassion. In Study 2, BMI, mindful eating, and self-compassion were explored using the Mindful Eating Behaviour Scale-Trait (MEBS-T; Mantzios, 2023b) focusing only on mindful eating behaviour using (1) sensory attention and (2) non-judgemental awareness with SOCS (Gu et al., 2020). The findings suggested only BMI was negatively associated with recognising and feeling from self-compassion, and no other significant relationships were found. In Study 3, BMI, mindful eating, and self-compassion were explored using MEBS-T (Mantzios, 2023b) and the original Self-Compassion Scale (SCS; Neff, 2003). The findings suggested that BMI and overidentification were negatively associated, whilst both sensory attention and non-judgemental awareness were positively associated with common humanity and mindfulness, and negatively associated with isolation. Moreover, non-judgemental awareness was also positively associated with self-kindness and negatively associated with overidentification. The findings from study 3 indicate that the relationship between mindful eating components of sensory attention and non-judgemental awareness with self-compassion is clear and consistent, which may in turn be translated into healthier eating behaviours. For example, research on both mindful eating and self-compassion has been found to be negatively associated with BMI, disordered eating symptomatology, fat and sugar consumption, and emotional eating (Mantzios et al., 2018; Taylor et al., 2015; Shaw and Cassidy, 2022).

The findings from Study 1 suggest that the decision-making component for mindful eating does not have a clear relationship to self-compassion. Previous versions of mindful eating scales, based on the early definitions of mindful eating that attempted to incorporate decision-making items in their measurements (Winkens et al., 2018) posed a risk of minimising the contribution of mindful eating, and more so of mindful eating behaviour. Furthermore, research on hunger and satiety has supported the idea that when one eats in response to their hunger cues, it can significantly reduce their caloric intake and promote healthier eating behaviours (Amin and Mercer, 2016; Ciampolini et al., 2013; Fukkoshi et al., 2015). Similarly, eating whilst distracted (e.g., using a smartphone, watching television, or engaging in a social context) can increase caloric intake (Blass et al., 2006; da Mata Gonçalves et al., 2019; La Marra et al., 2020). Therefore, it is reasonable to question whether including hunger, satiety, and distraction accurately measures mindful eating. These factors may inflate positive outcomes observed in other literature whilst neglecting the foundational elements that make eating mindful in the first place. Whilst awareness of one’s internal states of hunger and satiety may facilitate emotion-regulation through responding supportively, the behavioural implications are questionable when items do not purely reflect physiological hunger and satiety, but rather a belief or attitude (e.g., “I trust my body to tell me how much to eat”). The current research supports notions that separating mindful eating behaviour from decision-making for mindful eating may provide clarity in its association to other constructs, and in this case, with self-compassion. Ideally, mindful eating behaviour should encompass both sensory attention and non-judgement (as in the MEBS-T), and not just attentive eating (as in Winkens et al., 2018). If we, as researchers, are to assume that mindful eating is a derivative of secular mindfulness (Mantzios, 2023b), then the relationship to self-compassion should be unidimensional and consistent on all fronts, as demonstrated in Study 3.

Beyond mindful eating, the cooperative effects of combining mindful eating with self-compassion for positive behaviour change underscore the necessity of critically identifying and incorporating self-compassion that has a theory matching to measurement tools, and both interconnected to interventions. This aligns with Neff’s theoretical foundations and practical applications of self-compassion (Neff, 2003; Neff, 2003). Whilst elements such as “tolerating uncomfortable feelings,” as captured by the Sussex-Oxford Compassion for the Self Scale, may offer insights into emotion regulation (Gu et al., 2020), they fall short of providing a consistent, actionable framework for measuring, cultivating, and embodying a compassionate stance towards oneself, as demonstrated in Study 2. In other words, what is being measured in scales needs to translate into applied and actionable implications, and this critical point will enable the translation of self-compassion into effective practices and interventions.

Practical implications include behavioural interventions beyond direct links that have been previously explored. For example, an indirect link between mindful eating and internalised weight stigma may be explained through self-compassion (Fekete et al., 2021). A similar original implication for behavioural change could be the relationship to emotional eating, and how mindful eating indirectly influences eating in response to emotions through self-compassion (e.g., Zhang et al., 2021). For the first time, the appropriate tools create a momentum to explore behavioural (i.e., mindful eating) and emotional (i.e., self-compassion) paradigms of change, proposing clear pathways through which one may influence healthy eating: mindful eating via behavioural regulation, and self-compassion via emotion regulation.

A limitation of the present research that should be considered is that across the three studies, only a cross-sectional approach was employed. The cross-sectional nature of the present studies precludes conclusions along casual lines. Future research should utilise longitudinal experimental designs to explore the relationship between BMI, mindful eating, and self-compassion using MEBS-T and SCS with an intention to promote mindful eating and self-compassion. Furthermore, the present studies were conducted separately with participants varying across the three studies, future research could explore the current findings using the above scales on the same group of participants to consider individual differences. For example, controlling for age led to some slight differences in the relationship between BMI, mindful eating and self-compassion across the three studies, future research should use a diverse age group across one large study to gain a more thorough understanding on the role of age between BMI, mindful eating and self-compassion Additionally, the gender distribution across the first two studies was unequal, which may have had a potential influence on the current findings. For example, research has found mindful eating and self-compassion to differ between genders, with males scoring higher than females (Putri et al., 2024; Yarnell et al., 2015). Moreover, the present studies did not collect any information on educational or socio-economic background of the participants, which may also have had a potential influence of the current findings, as some research suggests socio-economic status may have a negative association with self-compassion (Parihar et al., 2024), whilst higher education could be associated with greater self-compassion (Bluth et al., 2020). Given these findings, future research should aim for a more equal gender distribution sample, and the inclusion of diverse demographic information. Importantly, the current studies used BMI as a biometric variable; however, given the ongoing discussions on the limitations of BMI as a health indicator (e.g., Wu et al., 2024), such as assessing excess fat, and differences in body build and ethnicity, future research should consider using alternative measures, such as body composition to allow for a more inclusive measure.

To conclude, this research involved three studies examining the relationships between BMI, mindful eating, and self-compassion using various psychometric tools. Study 1 faced challenges in clarifying the connections between mindful eating behaviour and decision-making components using MEBS and the SOCS. Consequently, Study 2 explored the MEBS-T with SOCS, but did not reveal significant insights into how mindful eating behaviour and self-compassion are related. Study 3 utilised the original Self-Compassion Scale (SCS), and found that sensory attention and non-judgemental awareness were positively associated with the positive aspects of self-compassion and negatively associated with the negative aspects. Future research should consider using both the MEBS-T and the original SCS to better understand how self-compassion interacts with mindful eating and to develop more effective practices that are aligned with mindfulness-based constructs and interventions for positive eating behaviour change.

## Data Availability

The raw data supporting the conclusions of this article will be made available by the authors, without undue reservation.
